# The modification and evaluation of an ELISA test for the surveillance of *Mycobacterium avium* subsp. *paratuberculosis* infection in wild ruminants

**DOI:** 10.1186/1746-6148-9-5

**Published:** 2013-01-09

**Authors:** Mathieu Pruvot, Taya L Forde, Jillian Steele, Susan J Kutz, Jeroen De Buck, Frank van der Meer, Karin Orsel

**Affiliations:** 1 Faculty of Veterinary Medicine, University of Calgary, 3330 Hospital Drive NW, Calgary, AB T2N 4N1, Canada

**Keywords:** Bison, Caribou, ELISA, Elk, Evaluation, *Mycobacterium avium* subsp. *paratuberculosis*, Sensitivity/specificity, Serology, Validity, Wildlife

## Abstract

**Background:**

Enzyme-linked immunosorbent assay (ELISA) is often used to test wildlife samples for *Mycobacterium avium* subsp. *paratuberculosis* (MAP) infection. However, commercially available kits are only validated for use with domestic ruminant species. A literature review was performed to document the current use of MAP serum ELISA in wild and semi-domestic ruminants. We then modified and evaluated a commercial ELISA kit (IDEXX *Mycobacterium paratuberculosis* Antibody Test Kit) for use with species for which it was not originally developed: elk (*Cervus elaphus*), bison (*Bison bison*) and caribou (*Rangifer tarandus*). We tested the affinity of different conjugates for immunoglobulin G (IgG) isolated from these species, performed checkerboard tests to determine the optimal dilutions of samples and conjugates, and established cut-off values using two different methods: a Receiver Operational Curve on a panel of known samples for elk, and an alternate method involving a panel of unknown serum samples for the three species.

**Results:**

We found that the anti-bovine conjugate included in the IDEXX ELISA kit has limited affinity for elk, bison, and caribou IgG. Protein G showed good affinity for IgG of all three species, while anti-deer conjugate also bound elk and caribou IgG. Using Protein G with elk serum, a cut-off sample-to-positive (S/P) value of 0.22 was selected, resulting in a sensitivity and specificity of 73% and 90%, respectively, whereas, using an anti-deer conjugate with elk serum, an S/P cut-off value of 0.29 gave a sensitivity of 68%, with 100% specificity. Cut-off values for bison and caribou using the Protein G conjugate were 0.17 and 0.25 respectively.

**Conclusions:**

Due to incomplete reporting and a lack of test validation, it is difficult to critically appraise results of many sero-surveys that have previously been done for MAP in wildlife. Commercial ELISA kits may have limited or no capacity to detect antibodies from species other than for which they were developed. In order to generate reliable test results, it is essential to evaluate the test and perform modifications if deemed necessary. Despite the challenges inherent to wildlife diagnostics, we have shown that several methods can be used to improve confidence in test results.

## Background

*Mycobacterium avium* subsp. *paratuberculosis* (MAP) causes granulomatous enteritis in ruminants that can lead to weight loss and emaciation, as well as diarrhoea in certain species [1]. MAP has been detected in wild ruminant species worldwide, and it is thought that all ruminant species are susceptible to MAP infection [2]. Although clinical disease, known as Johne’s disease, has only occasionally been described in wildlife, its impact may be under-estimated due to incomplete knowledge of the clinical progression of MAP infection in these species, non-specific clinical signs, and limited testing in these populations.

Determining the infection status of wildlife is important to reduce the disease risks associated with translocation, to certify infection status for importation/exportation purposes, to limit sources of infection for humans and livestock, and to establish baseline values for future monitoring or surveillance [3]. For the detection of current or prior MAP infection in wild ruminants, serological methods such as enzyme-linked immunosorbent assay (ELISA) may be preferred over alternative methods such as faecal culture, as ELISA is less expensive, fast and easy to perform, and collected serum can be used to screen for other infections.

Serum ELISA kits marketed for the detection of MAP-specific antibodies in domestic ruminant species are generally not validated for use with sera from other species [2]. It is unlikely that these tests give equivalent results for other, even closely related species [3]. Inter-species variations in test outcomes are due in part to the specificity of certain reagents included in commercial kits; for example, the labelled secondary antibodies or conjugates may have varying capacity to bind immunoglobulin G (IgG) from different species [4]. The level of non-specific binding of serum proteins to components of the ELISA assay may also be difficult to predict, and can potentially result in a reduction of the signal-to-noise ratio, which is the ratio of the optic density (OD) of a positive control and a negative control at a given sample and conjugate concentration [5]. Kits generally recommend cut-off values to classify samples as ‘negative’, ‘positive’ or ‘suspect’. Cut-off values are selected based on a set of parameters, in particular the species being tested, the target condition (i.e. infected, infectious, clinically diseased), and the testing objectives (e.g. demonstrating freedom from infection, estimating population-level prevalence, etc.) [3]. When using a commercial kit for wildlife samples, changes in these parameters need to be reflected by adapting the cut-off values.

Guidelines for conducting a complete diagnostic validation have been thoroughly outlined [6,7], and detailed recommendations for the design and reporting of diagnostic evaluation studies for chronic diseases have been developed [8]. However, it is not always possible or appropriate to undertake such a full evaluation in wildlife studies due to time and budget constraints as well as sample availability, in particular accessing known positive and negative controls. In these cases, certain modification and evaluation steps may reduce the level of uncertainty in the test results if a complete validation is impossible.

The objectives of this paper were first, to assess current practices in testing and reporting of MAP serum ELISA for wildlife samples, and second, to modify and evaluate a commercial ELISA kit (IDEXX *Mycobacterium paratuberculosis* Antibody Test^a^, hereafter referred to as the IDEXX kit) for detecting current or prior MAP infection in elk (*Cervus elaphus*), bison (*Bison bison*) and caribou (*Rangifer tarandus*).

## Methods

### Literature review

A systematic search of peer-reviewed articles and proceedings related to the development or use of ELISA tests in wildlife was performed. The keywords “serology”, “ELISA”, “wildlife”, “ruminant”, “cervid”, “deer”, “elk”, “bison”, “caribou”, “rangifer”, “diagnostic”, “paratuberculosis” and “Johne’s disease” were used in PubMed, ISI Web of Knowledge, ScienceDirect, and Google Scholar in January 2012. Papers found during the initial search were carefully examined for additional relevant citations. We included any papers that used MAP ELISA to test for naturally infected animals in ruminant species other than cattle, sheep and goats; experimental infections were excluded. Among the papers that fit the above-mentioned selection criteria, the following information was collected: type of ELISA (commercial kit, in-house test, modified test), modifications to the materials or procedure, and reporting of cut-off values and test performance.

### Conjugate affinity

In order to determine which conjugates could be appropriate for testing elk, bison, and caribou sera, we first evaluated the binding affinity of the anti-bovine conjugate included in the IDEXX kit^a^ to purified IgG from each species; cattle IgG were included for comparison. Protein G^b^, a recombinant bacterial protein that binds IgG from a wide variety of species, was also tested for all four species. A second anti-bovine conjugate^c^ (hereafter referred to as KPL anti-bovine) was tested against cattle and bison, and an anti-deer conjugate^d^ against elk and caribou as more specific alternatives.

For each species, IgG was purified from a pooled serum sample of four individuals, using the Melon Gel IgG Spin Purification Kit (Thermo Scientific, Pierce Biotechnology, Rockford, Illinois, USA), following manufacturer’s instructions. The protein concentration of each purified IgG product was determined using the Bradford titration method (Bradford Reagent, Sigma-Aldrich, St. Louis Missouri, USA). The purified IgG products from the four species were adjusted to a concentration of 400 μg/ml by addition of phosphate-buffered saline (PBS, pH = 7.2). Eight two-fold serial dilutions of the purified IgG product were made in a coating buffer (50 mM sodium carbonate pH = 9.6), from a starting concentration of 2 μg/ml. Subsequently, 100 μl of each dilution of purified IgG was incubated on an Immulon 4 HBX microtitre 96-well plate (binding capacity: 400–500 ng IgG/cm^2^; coefficient of variation <5%) for 2.5 hours at 37°C. Wells were washed three times with washing buffer (Phosphate-buffered saline solution containing 0.05% Tween20, hereafter called PBS-T), and blocked with 1% Bovine Serum Albumin (BSA) in PBS-T (300 μl per well) for one hour at 37°C. To ensure that there was no interaction between the conjugate and the blocking reagent, and to verify that a 1% solution was an optimal blocking concentration, control wells with different concentrations of the blocking solution (0.5%, 1% and 2.5% BSA) were included on the plate.

After repeating the previous wash protocol, 100 μl of the conjugate being tested was added to each well and incubated for 30 minutes at room temperature. Preliminary testing was conducted to determine the conjugate concentrations that would give an optical density (OD) of approximately 2.0 for at least one of the species being tested in order to obtain a sufficient range of ODs allowing between-species comparisons. These were determined to be 1:12000, 1:20, 1:100, 1:500 for Protein G, IDEXX kit anti-bovine, anti-deer and KPL anti-bovine, respectively, diluted in PBS-T 0.1% BSA (data not shown). Following incubation with the conjugate, the plate was washed three times, and wells were revealed by the addition of 100 μl of Tetramethyl benzidine (TMB) included in the IDEXX kit. The reaction was stopped after five minutes with the “stop solution” provided in the kit. The OD of each well was measured using a spectrophotometer at 450 nm (Expert 96, ASYS). We plotted the OD on the y-axis and the serial dilutions of IgG on the x-axis, and the area under the curve (AUC) was calculated (Excel, Microsoft Office 2010) as an index of binding affinity for comparison between species within each conjugate.

### Optimization

A checkerboard test (CBT) was performed using Protein G with all three species, as well as anti-deer for elk and caribou, to find the optimal concentrations of sample and conjugate that would provide the best signal-to-noise ratio. Appropriate ranges of serum and conjugate dilutions were identified by preliminary testing (data not shown), and all combinations within these ranges were tested with a positive and negative control sample, using a separate microtitre plate for each. For both bison and elk, high sero-reactors (based on preliminary testing using Protein G) were selected for use as positive controls from among individuals that had tested positive for MAP by either faecal or tissue culture. For these two species, negative control samples were available from farmed herds that had repeatedly tested negative by faecal culture over several years. For caribou, it was not possible to obtain samples from animals of known infection status; however, serum from an animal with a high MAP antibody titre was available, and this was used as a positive control [9]. Serum from a caribou with a low OD on preliminary testing was selected as a negative control in the CBT.

A 96-well microtitre plate provided in the IDEXX kit was used for each of the positive and negative control samples. These plates are pre-coated with MAP antigen. Eight two-fold dilutions of the sample were plated onto eight columns, starting at a 1:5 dilution. Samples were diluted in the sample diluent provided in the IDEXX kit, allowing the *M*. *phlei* adsorption step to partially remove cross-reacting antibodies [10]. After plating the samples, manufacturer’s instructions were followed until conjugate was added. At this step, eight two-fold dilutions of the conjugate were prepared in order to include approximately four dilutions above and below the concentration recommended by each conjugate manufacturer. Conjugate dilutions ranged from 1:500 to 1:64000 for Protein G and from 1:125 to 1:4000 for anti-deer. Each conjugate dilution was plated onto one of the eight rows of the assay plate. Wells containing conjugate diluted in PBS-T 0.1% BSA instead of the conjugate diluent from the IDEXX kit were included as a control for non-specific binding. The final steps of the ELISA were performed according to the manufacturer’s instructions, except for the length of revelation with the TMB substrate when using the Protein G conjugate, which based on visual observation was judged to be optimal at 5.5 minutes +/− 30 seconds.

The interpretation of the checkerboard test was done according to Crowther et al. [5]. The negative CBT was used to find the acceptable range of conjugate dilutions that minimized the background noise, i.e. the OD of a negative sample. On the positive CBT, we selected the range of conjugate dilutions that gave appropriate titration curves, i.e. the curve displayed a sigmoid shape from the highest to the lowest sample dilution, reaching the level of background signal. The final conjugate concentration was selected based on the overlap between these two ranges. To select the sample dilution, the binding ratio (OD positive/OD negative) was calculated for each combination of conjugate and sample dilution. The sample dilution was chosen to maximize this ratio, and as a secondary criterion, minimize the amount of serum required.

### Determination of cut-off value and sensitivity/specificity

#### Samples from animals of known infection status

The optimal way to establish cut-off values is to test a panel of samples from animals of known infection status [6]. This was possible for both elk and bison, as reference samples were available. For elk, the reference panel consisted of 20 negative and 22 positive samples. Negative samples were from individual farmed elk that tested negative to either faecal or tissue culture and serology done at the Johne’s Information Center, University of Wisconsin, Madison. These elk were from herds in Montana, USA that had no clinical evidence of Johne’s disease and were negative by repeated serum ELISA testing over several years.^e^ Positive samples were from individuals that tested positive by ELISA and by tissue and/or faecal culture at this same diagnostic centre, and were from herds with clinical cases of Johne’s disease. For bison, the panel contained 20 negative and five positive samples. Negative samples were from individual free-ranging bison that were negative by faecal culture, from herds with no evidence of infection over a five-year testing period. Positive samples were from clinically affected animals confirmed to be infected by tissue culture and that tested positive by ELISA and agarose gel immunodiffusion (AGID). These control samples were tested with the IDEXX kit using the optimal concentrations of sample and conjugate defined in the previous step. Protein G was tested for both species, and anti-deer was also tested for elk. Sample-to-positive (S/P) ratios were calculated for each sample using the IDEXX kit positive and negative controls as the reference (S/P = [Sample-RefNeg]/[RefPos-RefNeg]). From the results obtained from these panels, the optimal cut-off value, sensitivity and specificity were determined using ROC analysis, calculated using STATA 11.2 (StataCorp, Texas, USA).

#### Alternative cut-off calculation

As a panel of reference samples was not available for caribou, an alternative means for defining a cut-off was used. This method was also used for elk and bison to provide a comparison with the reference panel. For each species, a bank of serum from individual animals of unknown infection status, independent from the panel samples, was available. Sera from adult caribou (n = 135) were obtained from herds in western Greenland (provided by C. Cuyler of the Greenland Institute of Natural Resources) and northern Quebec, Canada (provided by S.D. Côté of Université Laval). These samples were collected between 2008 and 2009 through the CircumArctic Rangifer Monitoring and Assessment Network [11]. A total of 312 serum samples from yearling and adult elk were obtained from ten herds in western Alberta, Canada, through capture efforts of multiple projects occurring in the region between 2007 and 2012 (Montane Elk Research Project, Parks Canada, University of Alberta). Serum samples from 130 individual adult bison were collected by various collaborators from northern Alberta and British Columbia, and the Northwest Territories, Canada, between 2007 and 2011. The approach used to define the cut-off value and test performances for these samples was modified from Desquesnes et al. 2009 [12]. Briefly, the samples were tested according to the conditions determined in the previous steps, and S/P ratios calculated using the controls included in the IDEXX kit (to facilitate the use and reproducibility of these findings in other laboratories). Positive and negative controls from the species being tested were also included on each plate. The cut-off value suggested by the kit for negative samples (S/P = 0.3) was taken as the preliminary cut-off value for selecting presumed negative samples. The mean S/P value from this presumed negative pool was calculated, and a new cut-off value was defined (Mean S/P Neg + 3 standard deviations (SD)).

## Results

### Literature review

The literature search yielded a total of 60 papers; 29 were retained as relevant to the scope of this article [9,13-41], four of which were proceedings. Ten papers reported the use of in-house ELISA, 14 used commercial kits, and one article made a comparison of an in-house procedure and a commercial kit. It is noteworthy that in four papers it was impossible to identify which kit or procedure was used, and in some publications the procedure and modifications were insufficiently documented to allow for the experimental conditions to be reproduced. Of the studies that used commercial kits, 12 did not perform any modifications to account for testing a different species. Overall, only five papers reported any kind of test evaluation or referred to studies that had done so. Eleven papers failed to discuss the cut-off value used to distinguish between positive and negative results. Only one paper reported the sensitivity and specificity values of their modified test for the species for which the test was applied [23].

### Conjugate affinity

**Figure 1:**
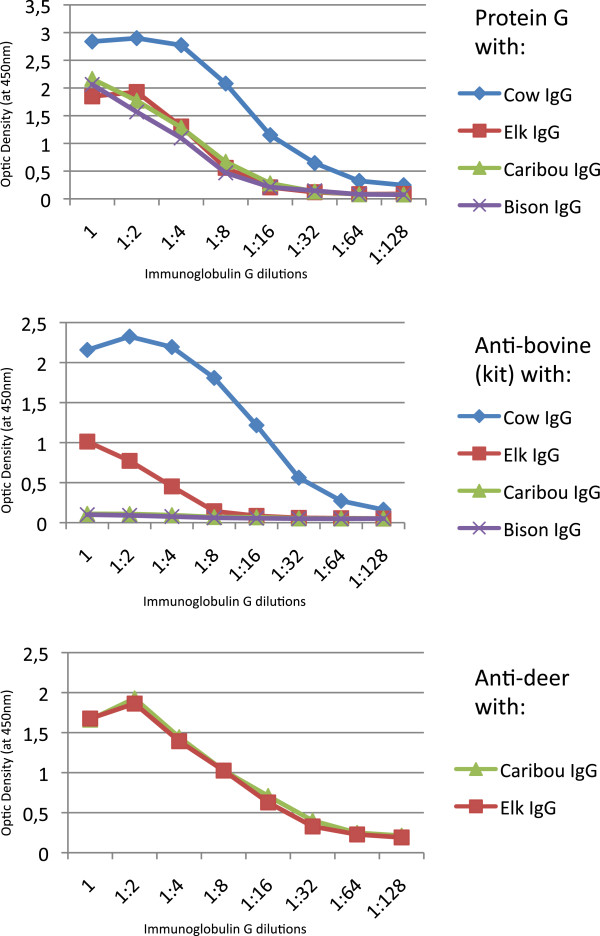
**Binding affinity of Protein G and anti**-**bovine conjugate for cow**, **elk**, **caribou and bison IgG**, **and affinity of anti**-**deer conjugate for caribou and elk IgG.** Dilutions are given as fractions of an initial IgG solution at 2 μg/ml.

The affinity of the IDEXX kit anti-bovine conjugate was high for cow IgG (area under the curve (AUC) = 9.5), low for elk (AUC = 2.1), and insignificant for bison and caribou (AUC = 0.5; Figure 1). Similarly, the KPL anti-bovine conjugate had a very high affinity for cow IgG (AUC = 23.0) and a much lower affinity for bison IgG (AUC = 4.7). Protein G had a comparable affinity for elk (AUC = 5.2), caribou (AUC = 5.3) and bison (AUC = 4.6), but a higher affinity for cow IgG (AUC = 11.4). Finally, the anti-deer conjugate showed comparable affinity between elk (AUC = 6.4) and caribou (AUC = 6.7). No background signal was detected in any of the control wells coated with blocking solution.

### Optimization

The optimal conjugate and sample dilutions determined for each species are presented in Table 1. No significant difference in OD was observed between the conjugate diluted in PBS-T versus the conjugate diluent provided in the IDEXX kit, ensuring the absence of non-specific binding between the kit conjugate diluent and the new conjugates.

**Table 1:** Checkerboard test results

	**Elk**	**Caribou**	**Bison**
Protein G dilution ^(a)^	1:16000	1:8000	1:16000
Anti-deer dilution	1:250 ^(b)^	1:175 ^(c)^	NT
Sample dilution using Protein G	1:30	1:20	1:160
Sample dilution using anti-deer	1:30	1:5	NT

(a) Concentration not available from manufacturer.

(b) 0.40 μg/ml.

(c) 0.57 μg/ml.

Optimal dilutions of Protein G and anti-deer conjugates when used with serum from elk, caribou or bison, and optimal species-specific serum dilutions for use with the IDEXX *Mycobacterium paratuberculosis* Antibody Test. NT: not tested.

### Determination of cut-off value and sensitivity/specificity

#### Samples from animals of known infection status

ELISA test parameters for elk serum using either the Protein G or anti-deer conjugate are given in Table 2. Since Protein G and anti-deer conjugates resulted in the correct classification of a similar number of animals, and since Protein G is more readily available to end-users, we selected this conjugate to proceed with further testing. A cut-off value could not be established using the panel of known bison samples due to the nature of the positive samples in the panel, which were from animals with advanced clinical disease. These sera had very high S/P ratios, and were unlikely representative of subclinically infected animals; we would not expect animals at earlier stages of infection to have such high antibody concentrations. Given this biased distribution of S/P ratios in our positive control group, we determined that this method would result in an inappropriate cut-off for our target population.

**Table 2:** **ELISA test parameters when using protein G or anti**-**deer conjugates with elk serum**

	**S**/**P cut**-**off**	**AUC** [**95**% **CI**]	**Se** [**95**% **CI**]	**Sp** [**95**% **CI**]	**PPV** [**95**% **CI**]	**NPV** [**95**% **CI**]
Protein G	0.22	0.81 [0.65; 0.91]	73% [50; 89]	90% [68; 99]	89% [65; 99]	75% [53; 90]
Anti-deer	0.29	0.85 [0.71; 0.95]	68% [45; 86]	100% [83; 100]	100% [78; 100]	74% [54; 89]

Sample-to-positive (S/P) ratio cut-off values as determined by Receiver Operational Curve (ROC) analysis, the area under the curve (AUC), and the resulting sensitivity (Se), specificity (Sp), positive predictive value (PPV) and negative predictive value (NPV).

#### Alternative cut-off calculation

Of the 312 elk samples of unknown status, 308 were presumed negative as defined by the preliminary cut-off of S/P = 0.3. S/P ratios of the presumed negative samples were averaged (m = 0.066, SD = 0.039) and a new cut-off value was calculated as m + 3 SD = 0.18. Using this method with bison sera, 129 of 130 samples of unknown status were presumed negative with the preliminary cut-off. After calculating the average S/P ratio (m = 0.039) and standard deviation (SD = 0.042) of these samples, the new cut-off was defined as 0.17. Among 135 adult caribou serum samples, 134 samples were presumed negative, yielding a final cut-off of 0.25 (m = 0.083, SD = 0.054).

## Discussion

### Literature review

We found that ELISA is commonly used for testing for MAP infection in different wild and semi-domestic ruminant species, but discrepancies exist in terms of the quality of the data reported. In many of the papers we reviewed, the methods were incompletely described, limiting the ability of the reader to reproduce the test conditions or to critically interpret the results of these studies. Despite inherent challenges in wildlife diagnostics, appropriate test modification and validation steps are necessary. We have presented a range of experiments that can be undertaken to reach this objective.

### Conjugate affinity

We found that the choice of conjugate has an important impact on the test performance due to species-associated differences in affinity. Although it was impossible to directly compare the binding affinity of the different conjugates due to the inaccessibility of information about the concentration of these products (proprietary information), it was possible to compare the affinity of each conjugate between species. Protein G would be a suitable conjugate for IgG from any of the three species of interest, and anti-deer is an appropriate alternative for elk and caribou samples. The low affinity of the IDEXX kit anti-bovine conjugate for elk, bison and caribou immunoglobulin makes it inappropriate for testing samples from these species.

In this study, the binding affinity of Protein G for elk and caribou was similar, although lower than it was for cattle; the same trend was previously shown for red deer (*Cervus elaphus*) and reindeer (*Rangifer tarandus*) in comparison to cattle [37]. Our results differed slightly from those reported by Kramsky et al., who found that the Protein G binding affinity was similar for bison and cattle, although similarly to this study, they found that elk had a lower binding affinity than cattle [4]. Our findings also confirmed these authors’ observation that it is impossible to infer binding affinity between closely related species.

### Optimization

Table 1 contains the recommended sample and conjugate dilutions for using the IDEXX kit with elk, caribou and bison sera. Aside from using these modified dilutions and alternate conjugates, we recommend that the protocol be followed according to the manufacturer’s recommendations.

We observed considerable variations in test results associated with the duration of incubation with the Tetramethyl benzidine (TMB) substrate; it is important that this time be kept consistent between test development steps and sample runs. Even when keeping the incubation time consistent, there were small differences between runs of the panel of samples. This variability may in part be attributed to the very low concentration of Protein G used in our assay, as concentrations may be difficult to reproduce exactly between tests.

### Determination of cut-off value and sensitivity/specificity

It has been suggested that to establish a cut-off value and subsequently calculate the test accuracy with a high level of precision, a panel of 100 positive and negative control samples should be tested [3]. Other authors suggest using thousands for validation prior to commercialization [7]. Meeting these recommendations becomes even more challenging for wildlife when selection criteria for appropriate control samples are considered. In an ideal scenario, samples should reflect the target population in terms of the age and sex ratio, the geographic location, and the distribution of animals between different stages of infection [42]. They should also reflect the target condition that has been defined by the researcher; for example, the three target conditions that are described for MAP are infected, infectious, and affected [42].

Our panel of known-status elk samples included animals of all three target conditions: clinically affected; seropositive and tissue culture positive (infected); and seropositive and faecal culture positive (infectious). This panel is therefore compatible with our objective of detecting infected animals regardless of their disease stage. However, the proportion of animals in each category may not reflect what is seen in a free-ranging population. The high number of clinically diseased animals among the positive control elk samples in our study likely means that we overestimate this ELISA’s ability to detect infected animals. Due to the relatively small number of known elk samples in our panel, our sensitivity and specificity estimates had large confidence intervals. The panel of known bison samples were even less representative of the target population since all positive animals were clinically diseased, making inferences about test sensitivity and specificity for infected animals impossible. Another limitation of the panel of reference samples is that an ELISA test of unknown performance was used to confirm some of the negative control samples. The Johne’s Information Center used an older version of the IDEXX test that employed an anti-ruminant conjugate as opposed to an anti-bovine conjugate. Although we are certain that this test was capable of detecting positive animals, the sensitivity is unknown. If there were animals falsely classified as negative in our panel, the result would be an underestimation of the specificity of our modified test.

When recommended numbers of appropriate control samples are unavailable, a cut-off value may be established either by using a panel with fewer samples of known status (recognizing the limited precision of the estimates of sensitivity and specificity), or by using alternate methods for calculating a cut-off value (recognizing that they are imperfect approximations and lack robustness). A commonly-used alternative is to identify and test a group of confirmed uninfected animals and to use the mean S/P + 3 SD of this group as the cut-off [5]. In this paper, we illustrated the worst-case scenario in which no reference samples for the species of interest were available and a presumed negative group had to be created. With this method, the final cut-off value is very sensitive to variations in the initial cut-off value selected. However in our study, the S/P cut-off values calculated for elk using both a panel of known control samples (0.22) and this alternative method (0.18) were quite similar. Since this was the only method that could be used for establishing a cut-off value for bison and caribou sera, sensitivity and specificity estimates could not be generated for this ELISA test for these species.

Testing objectives are important for the selection of the cut-off value, as researchers may wish to prioritize either a higher sensitivity or specificity. Although not the scope of this paper, our end-goal of adapting ELISA for these different species was to use it as a screening method to identify individual animals that are more likely to be infected. This would be confirmed using more specific MAP diagnostic methods such as faecal culture. Based on this objective, we wanted to select a cut-off that would optimize both sensitivity and specificity and give the highest percentage of correctly classified samples. If this test were to be used as a more definitive diagnostic test, for example in a test-and-cull strategy, the cut-off for a “positive result” needs to be selected to give a very high specificity. Using the ROC curve from our panel of elk samples using the Protein G conjugate, the cut-off value that gave 100% specificity was 0.5. The range of S/P values between our screening cut-off and this cut-off for positive samples could be designated as “suspect” results (0.2 to 0.5). Alternatively, the anti-deer conjugate could be selected over Protein G at the recommended dilution if the test objectives required a higher specificity.

## Conclusions

In conclusion, despite the abundant literature that exists on test development and validation, diagnostic tests validated only for livestock species are frequently applied for wildlife samples. As we illustrated, unmodified commercial tests may fail to detect the immunoglobulins of other species. From a wildlife management perspective, this can have serious implications for epidemiological surveillance. In this study we adapted a commercial ELISA kit for use with other species: we evaluated the binding affinity of different conjugates to IgG of the target species, ensured the absence of non-specific binding after modifications, performed a checkerboard test to recommend optimal sample and conjugate dilutions, and determined appropriate cut-off values. Such modification and evaluation steps enable valuable data to be generated from serological surveys for MAP in wildlife that can be critically appraised and interpreted with greater confidence.

## Endnotes

^a^IDEXX *Mycobacterium paratuberculosis* Antibody Test, Westbrook, Maine, USA; manufactured by Institut Pourquier, Montpellier, France.

^b^HRP-recombinant-Protein G, Invitrogen, Camarillo, California, USA.

^c^Peroxidase-Labeled Antibody to Bovine IgG, Kirkegaard & Perry Laboratories (KPL) Inc., Gaithersburg, Maryland, USA.

^d^Peroxidase-Labeled Affinity Purified Antibody to Deer IgG, KPL Inc.

^e^The Johne’s Information Center used the IDEXX MAP ELISA kit manufactured in 2006 (different version than a.).

## Competing interests

The authors declare that they have no competing interests.

## Authors’ contributions

MP and TF contributed equally to the study design and in drafting the manuscript. MP took the lead on the assay evaluation and modification, as well as in evaluating the test performance. TF assisted with the assay evaluation and modification steps, and formatted the manuscript. JS assisted in the study design, in carrying out the serologic testing, and provided feedback on the manuscript. SK, JDB, FVDM and KO participated in the study design and provided critical feedback on the manuscript. All authors read and approved the final manuscript.
